# Evaluating Food Additives Based on Organic and Inorganic Salts as Antifungal Agents against *Monilinia fructigena* and Maintaining Postharvest Quality of Apple Fruit

**DOI:** 10.3390/jof9070762

**Published:** 2023-07-19

**Authors:** Nadia Lyousfi, Ikram Legrifi, Nabil Ennahli, Abdelali Blenzar, Said Amiri, Salah-Eddine Laasli, Nadia Handaq, Zineb Belabess, Essaid Ait Barka, Rachid Lahlali

**Affiliations:** 1 Phytopathology Unit, Department of Plant Protection, Ecole National d’Agriculture de Meknès, Km 10, Rte Haj Kaddour, BP S/40, Meknès 50001, Morocco; nadia.lyousfi@usmba.ac.ma (N.L.); ikramlegr@gmail.com (I.L.); nabilennahli@gmail.com (N.E.); laaslisalaheddine@gmail.com (S.-E.L.); samiri@enameknes.ac.ma (S.A.); 2 Laboratory of Plant Protection and Environment, Faculty of Sciences, Moulay Ismail University, Zitoune, Meknès 11201, Morocco; ablenzar@yahoo.fr; 3 Laboratory of Functional Ecology and Environmental Engineering, Sidi Mohamed Ben Abdellah University, Route d’Imouzzer, Fez 30000, Morocco; 4 Equipe de Recherche, Valorization et Protection des Plantes, Laboratoire de Biologie d’Environnement et Developpement Durable, Ecole Normale Supérieure de Tétouan, Abdelmalek Essaadi University, Tetouan BP 209 Martil, Martil 93150, Morocco; n.handaq@uae.ac.ma; 5 Plant Protection Laboratory, Regional Center of Agricultural Research of Meknes, National Institute of Agricultural Research, Km 13, Route Haj Kaddour, BP.578, Meknes 50001, Morocco; zineb.belabess@inra.ma; 6 Unité de Recherche Résistance Induite et BioProtection des Plantes-EA 4707—USC INRAe1488, SFR Condorcet FR CNRS 3417, Faculty of Sciences, University of Reims Champagne-Ardenne, 51687 Reims, France; ea.barka@univ-reims.fr; 7 Plant Pathology Laboratory, AgroBioSciences, College of Sustainable Agriculture and Environmental Sciences, Mohammed VI Polytechnic University, Lot 660, Hay Moulay Rachid, Ben Guerir 43150, Morocco

**Keywords:** food additives, *Monilinia fructigena*, apple, conservation, postharvest

## Abstract

A set of commonly used food additives was evaluated for their antifungal activity against the brown rot disease of fruits caused by the fungal pathogen *Monilinia fructigena*, which is one of the most economically important agents, causing important damage to pome fruits, such as pears and apples. The radial mycelial growth of the fungal pathogen was assessed in PDA amended with different concentrations (0.5, 2, 2.5, and 5%) of each additive. The results underlined that most of the additives displayed a significant inhibition of mycelial growth, with the extent of inhibition varying depending on the specific additive and concentration used. Five food additives showed high inhibition rates (above 88%), of which sodium bicarbonate, sodium carbonate, copper sulphate, and sodium hydroxide were the most effective, whereas ammonium carbonate, magnesium chlorite, and citric acid were the least effective. Interestingly, the coatings containing sodium bicarbonate, copper sulphate, and ammonium bicarbonate significantly reduced the incidence of brown rot disease in apples, but other additives were not effective, such as ammonium carbonate and magnesium sulphate. The anhydrous sodium sulphate used at a concentration of 2%, was found to be one of the least effective additives, with a reduction rate of 20%. Subsequently, food additives showing good growth inhibition rates and reduction in disease severity were then tested in semi-commercial trials at temperatures of 4 °C and 22 °C. The results indicated that these additives demonstrate effectiveness in controlling *M. fructigena* at specific concentrations, and lower temperatures (4 °C) can improve the efficiency of the control measures. In addition, the selected food additives exhibited significant antimicrobial activity against *M. fructigena*, suggesting their application as a promising alternative for managing brown rot disease in apple fruits.

## 1. Introduction

The agricultural sector plays a fundamental role in Morocco’s economic activities. Likewise, it makes a substantial contribution to the nation’s development since it accounts for 20% of the gross domestic product and employs around 40% of the working-age population [1]. The apple tree (*Malus domestica*) is a highly significant agricultural species in Morocco. Currently, it covers 32,000 ha, accounting for nearly 25% of the total fruit-bearing surface of the Rosaceae family. The cultivation of apple trees has experienced rapid growth, driven by a thriving market, an expanding range of varieties, and a dynamic industry [2]. The main areas of apple plantations in Morocco are Meknes, Midelt, Khenifra, Haouz Marrakech, Fes, and Ouarzazate [2]. The Royal Gala variety is largely planted in cropping areas, such as Imouzzer-Kandar [3]. Apple fruits have worldwide demand, not limited to Morocco, making *Malus pumila* Mill a globally significant tree fruit worldwide [4]. It is a nutritionally important crop that is primarily consumed as fresh fruit, sought after by consumers for its flavor and nutritional qualities. Many countries have emerged as leaders in the production of apple fruit. The Russian apple market generates about 2.6–4.3 million tons of apples per year [5]. However, a limited percentage of apples is processed into jellies, cooked slices, and juices. Many fungi, including *Monilinia fructigena*, which causes brown rot disease and large economic losses even during storage, can infect this fruit and produce fungal infections. Pome and stone fruits are susceptible to brown rot disease, which can also lead to leaf blight and blossom [6]. Apple fruits are affected by different species of brown rot, either by *M. fructigena* or *Phytophthora syringe*, as described by Giraud and Bompeix [7]. Brown rot is among the most serious diseases of apples during storage and can lead to significant losses in the orchard during preharvest. For example, in the UK, losses of apples due to brown rot disease caused by *M. fructigena* are estimated to be up to 22% [8].

In general, postharvest fruit loss caused by plant pathogenic fungi accounts for more than 50% of all fruit and vegetable agricultural products [9]. Fungi of the genus *Monilinia* are present worldwide and cause economically important damage [10]. Several *Moniliia* species responsible for brown rot disease [11] affect pome and stone fruits economically, both in pre- and postharvest conditions. Brown rot, caused by three species of the *Monilinia*, mainly *M. laxa*, (Aderh & Rulh) Honey, *M. fructigena* Honey in Whetzel, and *M. fructicola* (Win) Honey, is the major disease in the European Mediterranean stone fruit production regions, including Spain [12]. The disease can reach a high incidence, leading to significant losses during storage [13,14]. *M. fructigena*, one of the three species, is one of the most important fungal pathogens that causes brown rot in apples and heavily affects fruit production [13]. The pathogen is frequently found in Europe and Asia [10]. For instance, *M. fructigena* is the predominant causal agent of brown rot disease in Serbia (76.48%) [15]. The majority of fruit losses during storage are caused by diseases due to different microbial infections, leading to 25–50% fruit deterioration [16]. The disease is mainly controlled by synthetic fungicides. However, fungicides are harmful to human health and affect negatively the environment [17]. In addition to satisfying the increasing demand for food supplies, the food industry sector has prioritized consumer health and safety [18]. In this regard, the only available treatment for managing this disease is through field fungicide spraying. However, it is unfortunate that no chemicals are allowed in the EU after the harvest of stone fruit [19]. Therefore, the use of these synthetic pesticides should be reduced.

Many chemicals classified as GRAS have been used to extend fresh fruit shelf life during storage, such as bicarbonate, calcium carbonate, and silicate [17]. These organic and inorganic salts were also found to be quite promising [20]. To increase the effectiveness of electrolysis and replace the use of NaCl, which is known to produce corrosive by-products that harm equipment, operators, and consumers, several organic and inorganic salts were evaluated in the past. Among these salts was sodium bicarbonate (NaHCO_3_), a food additive which is used in various commodities [13]. It has also been reported to be effective in preventing postharvest diseases in a wide range of fruit. It is widely used in the food industry as a food additive and is allowed with no restrictions for many applications under European and North American regulations [21]. Moreover, SBC is a very attractive alternative because it is readily available, inexpensive, and has little risk of phytotoxicity at low concentrations (1–4%) [12]. Interestingly, trisodium phosphate (TSP), which is defined as a GRAS substance by the US Food and Drug Administration, can reduce brown rot disease and inhibit *M. fructicola* growth [17]. This inorganic compound is highly soluble in water and produces alkaline solutions. 

Our study aimed to evaluate the innovative biological and integrated approaches to control brown rot disease in apples by investigating the impact of different food additives (GRAS) such as magnesium, sodium, sulphate anhydrous, ammonium carbonate, ammonium bicarbonate, copper sulphate, and other salts on the development of *M. fructigena* both in in vitro and in vivo trials. Therefore, we studied the effect of salts on the mycelial growth of the pathogenic fungus, the inhibition of spore germination, and the effect of different treatments on germ tube elongation. In the in vivo experiments, the severity and incidence of the disease on artificially infected fruits as affected by salt treatments were assessed. In addition, different indices of fruit quality, such as weight loss, titratable acidity, total soluble balances, and firmness were recorded. The treatments showing a high inhibition rate and low severity were then evaluated in a semi-commercial trial at two different temperatures, 22 and 4 °C.

## 2. Materials and Methods

### 2.1. Fungal Pathogen

The *M. fructigena* VPBG used in this study was isolated from cherries in Serbia and identified as previously described [22]. The fungal pathogen was sub-cultured on PDA (Biokar Diagnostics, Zac de Ther, France). The culture was then incubated for seven days at a temperature of 25 °C in a dark environment. A 10–day-old fungal colony was flooded with 5 mL of sterile distilled water (SDW) containing Tween-20 (0.05%) to prepare the conidial suspension. The conidial suspension was recovered by scraping the medium’s surface using a sterile Pasteur pipette tip. The resulting suspension was filtered through autoclaved tissue layers to remove mycelial debris and the rest of the medium. The final concentration of the conidial suspension (4.5 × 10^4^ spores/mL) was adjusted using a Malassez hematocytometer (Roche, Meylan, France).

### 2.2. Fruit Samples

To assess the in vivo effect of salt treatments on brown rot disease, mature apple fruit (*Malus domestica*) belonging to “Ginger gold” were harvested from a field in Azrou (Morocco). Apple fruit with a uniform size and free from any diseases or disorders were carefully selected for the experiment. These apples were stored in a cold room at 4 °C to ensure their preservation and prevent any contamination or degradation before the experiment. Before the experiment, the apple fruits were surface sterilized by dipping them in a 2% sodium hypochlorite solution for 2 min, and then washed twice with SDW and air-dried for 1 h at room temperature [23,24].

### 2.3. Fungicidal Treatment

Difenoconazole was used as the referential fungicide for the in vivo tests. This fungicide is a sterol-inhibiting (SI) fungicide recently registered for use on grapes and other fruit. It belongs to FRAC Group 3, along with myclobutanil (Rally), tebuconazole (Elite, Tebuzol, Orius), and fenarimol (Vintage, formerly Rubigan). In this study, this fungicide was used at a concentration of 1 ppm.

### 2.4. Food Additives

The names, acronyms, molecular formulas, and molecular weights of the antimicrobial food additives used in this work are listed in Table 1. They are inorganic and organic salts classified as GRAS or food additives by the USA or EU legislation [25].

Laboratory reagent grade preservatives (99% minimum purity) were purchased from Sigma-Aldrich Chemie, Fluka Chemie AG (Buchs, Switzerland), Panreac Química S.L.U., or Merck KGaA (Darmstadt, Germany). Potassium silicate (PSi), as the commercial product Sil-Matrix^®^ (29% PSi), was purchased from the PQ Corporation (Valley Forge, PA, USA) [25,26].

### 2.5. Determination of the In Vitro Antifungal Activity of Food Preservatives

The potential of the food agents to inhibit the mycelial growth of *M. fructigena* was assessed on Petri dishes filled with PDA medium amended at 45–55 °C with sterile aqueous solutions of the respective antimicrobial agent. Each food preservative was prepared as a stock solution in various concentrations (0.5, 2, 2.5, and 5%) by dissolving the required quantity in SDW. The PDA media without food agents served as controls. The center of each Petri plate was inoculated with a 5 mm mycelial plug from the edge of a fresh 7–10-day-old colony of *M. fructigena* and incubated for 15 days at 25 °C in darkness. For each plate, the average of two perpendicular fungal colony diameters was recorded to determine the radial mycelial growth after 5- and 15-day post-incubation periods. Three replicate plates were used for each preservative agent and concentration, and the trial was repeated twice over time. The results were expressed as an inhibition rate (%) of mycelial growth according to the formula described by Karaca et al. [26]:

Inhibition rate (%)=(Dc−Dt)/Dc×100

where Dc = average diameter of the fungal colony grown on PDA control plates and Dt = average diameter of the fungal colony grown on PDA amended plates with each preservative agent.

### 2.6. Observation of the Mycelium Growth of M. fructigena

After 15 days of incubation, the mycelial structure of the fungal pathogen under different treatments was examined using a light microscope (Ceti Microscopes NLCD-307B, Chalgrove, UK) with a magnification of (10 × 40). This test was conducted to reveal the effect of salts on the structure and morphology of the hyphae of *M. fructigena* [27].

### 2.7. Determination of Spore Germination and Germ Tube Elongation

The method used to study the effect of salts on spore germination and germ tube elongation of *M. fructigena* consisted of mixing the conidial suspension of the fungal pathogen with each salt’s concentration (0.5, 2, 2.5, and 5%) at equal volume (1v:1v). The control consisted of using the same concentration of conidial suspension without salts. All mixtures were incubated at 25 °C on sterile microcentrifuge tubes with agitation. Spore germination was observed under a light microscope for 24 h post-incubation periods. At least 100 spores within each replicate were examined at 400× *g* magnification. The spore was considered germinated if the length of the germ tube was longer than its smallest diameter. The following formula was used to calculate the inhibition rate of spore germination:

Inhibition rate of spore germination (%)=(A−B)/A×100

where A represents the number of germinated spores in the control plates and B represents the number of germinated spores in each food additive treatment [28]. The salts’ impact on the germ tube length was also assessed. Triplicates were evaluated for each biocontrol agent concentration [29].

### 2.8. Packinghouse Experiments

#### 2.8.1. In Vivo Effectiveness of Food Additives against Brown Rot Disease

To confirm the in vitro results of potential GRAS salts against the fungal pathogen *M. fructigena*, an in vivo trial was conducted on apple fruit. Apple fruits were surface sterilized in a solution of sodium hypochlorite as described above [16]. To assess the biocontrol efficacy of salts, apples were wounded twice at the equatorial zone using a stainless-steel rod; each wound was 3 mm in diameter and 4 mm in depth. Each apple was placed in a disinfected cylindrical plastic box (1L capacity). A piece of Whatman paper sprayed with SDW was placed in the bottom of each box to maintain the high humidity required for fungal growth and to prevent moisture loss from the fruit. Each apple wound received 50 μL of antimicrobial food additive before being inoculated 24 h later with 50 μL of the conidial suspension of *M. fructigena* at 4.5 × 10^4^ spores/mL. Wounds that received only SDW served as controls [29]. In addition, a fungicidal treatment based on difenoconazole (1 ppm) was used as a positive control. Plastic boxes containing inoculated apple fruit were then incubated in a growth room at 22 °C in darkness. This experiment was repeated twice over time with 10 apples (20 wounds) per treatment. The development of decay was monitored 5 and 10 days post-inoculation and the lesion diameters were recorded using a caliper. Disease severity was determined as lesion diameters (mm) in each additive treatment compared to the control treatments [26].

Also, the disease incidence (%) was calculated for each treatment according to the following formula [16]:

Disease incidence (%)= number of infected fruits/total number of fruits×100

#### 2.8.2. Fruit Quality Measurements on Treated Apple Fruit

##### Determination of Weight Loss (%)

The weight loss of individual apple samples was determined by weighing them before applying salt treatments (0 days) and after 10 days of storage at 22 °C. Results were expressed as the weight loss percentage relative to the initial value [30]. The weight loss was calculated according to the formula proposed by Salem et al. [31].

Weight loss (%)=(initial mass−mass at examined date)/initial mass×100

##### Total Soluble Solid Content (TSS)

Total soluble solids were determined using an Atago digital refractometer (Atago refractometer model N-50E, Bellevue, WA, USA), at standard temperature (20 °C), and the results were expressed in Brix percentage [30,31].

##### Determination of Fruit Firmness (N)

The firmness was measured using a texture analyzer device (Texture Analyzer, CT-3, Brookfield, VT, USA) by recording the maximum force (mN) record during compression of the specimen between the base and a flat cylindrical probe. The compression length and the probe speed were 3 mm and 1 mm/s, respectively [30,32].

##### Malic Acid (MA)

The titration approach was used to determine the MA. For this, apple juice from five apples of each treatment was blended with distilled water using a blender. The acidity of apples was determined via potentiometric titration, using 1 mL of diluted juice in 25 mL of distilled water. Two drops of 0.1% phenolphthalein solution were added to the solution as an indicator [33]. This solution was then titrated using 0.1 N NaOH until a pink color was observed. The calculated results were the percentage of malic acid per 100 g of fresh weight [34,35].

##### Maturity Index

The maturity index of apple fruits was assessed as previously described [36,37]. The index was calculated using the ratio between TSS (the total soluble solids) and MA (malic acid).

#### 2.8.3. Semi-Commercial Large-Scale Trial

This experiment is a mandatory conclusive step to confirm the efficacy of the selected food preservatives to control brown rot disease. This trial is typically conducted with fresh fruit that has been artificially inoculated with the specific pathogen to simulate real postharvest conditions and identify the most effective treatment for preserving apple fruit during storage. Among the 16 inorganic and organic salts tested in the semi-practical trials of the selected salts, five salts displayed low disease severity in in vivo experiments, and demonstrated the highest inhibition rates of mycelial growth. Healthy apple fruits were sterilized and wounded (3 mm in diameter and 4 mm deep) at four equidistant points using a needle, and then dipped for 2 min in different salt concentrations (0.5, 2, 2.5, and 5). Apple fruits dipped in SDW served as a control [16]. Apple fruits were placed in plastic bags (3 apple fruits/bag) with three replicates and incubated in the growth chamber at two different temperatures, 22 and 4 °C. After 24 h of the incubation period, apple fruits were inoculated by spraying a fungal spore suspension of *M. fructigena* (4.5 × 10^4^ spores/mL) and returned to the growth chamber at 22 and 4 °C for the rest of the test. The number of infected fruits was monitored every 5 days until apples in the control treatments were fully infected. The experiment was repeated twice over time. The disease incidence was calculated at ambient temperature (21 ± 2 °C), according to the formula of Zhang et al. [24]:

Disease incidence=number of diseased fruits/total number of fruits×100

### 2.9. Statistical Analysis

All experiments were conducted twice over time. Analysis of variance (ANOVA) was performed using SPSS statistical software (version 20). When the effect was revealed to be significant, the Tukey test was used for means separation at *p* ≤ 0.05.

## 3. Results

### 3.1. In Vitro Effect of Antifungal Activity of Food Preservatives

**Figure 1:**
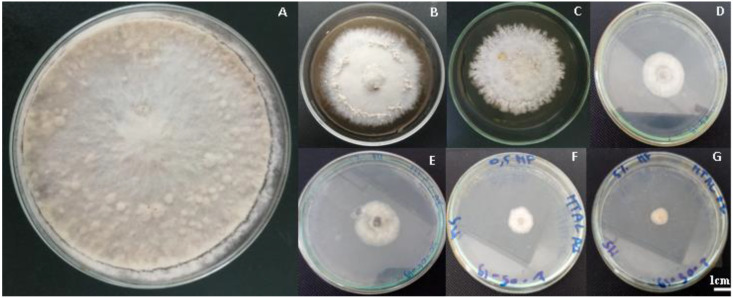
Colony morphology of *M. fructigena* as affected by the different antimicrobial food agents after 15-day incubation periods at 25 °C; (**A**) Control; (**B**) Magnesium chlorite (0.5%); (**C**) Sodium sulphate anhydrous (5%); (**D**) Ammonium carbonate (0.5%); (**E**) Ammonium bicarbonate (2%); (**F**) Copper sulphate (0.5%); and (**G**) Copper sulphate (5%).

The antifungal activity was determined based on the inhibition rate of mycelial growth of the fungal pathogen *M. fructigena* compared to the growth of this fungus on control plates without salt after 5 and 15 days of incubation at 25 °C (Figure 1, Table 2). Five days post-incubation, we observed that fungal growth remained completely inhibited in some plates, with more than 90% inhibition for copper sulphate. Significant differences between treatments were found, and the effect of each salt depended on the concentration applied (Table 2). Sodium bicarbonate, copper sulphate, ammonium bicarbonate, sodium carbonate, and sodium hydroxide were the most effective antifungal salts against *M. fructigena*. At a concentration of 2%, significant inhibition percentages exceeding 90% were observed. Notably, ammonium bicarbonate showed a 92.90% inhibition after just 5 days of incubation. Copper sulphate exhibited a complete inhibition at 100% at the same concentration, comparable to the effectiveness of fungicidal treatment. Sodium bicarbonate, sodium carbonate, and sodium hydroxide also displayed reductions exceeding 90% with inhibition rates of 91.92, 97.99, and 98.30%, respectively (Table 2).

### 3.2. Effect of Food Preservatives on the Mycelial Growth of M. fructigena

**Figure 2:**
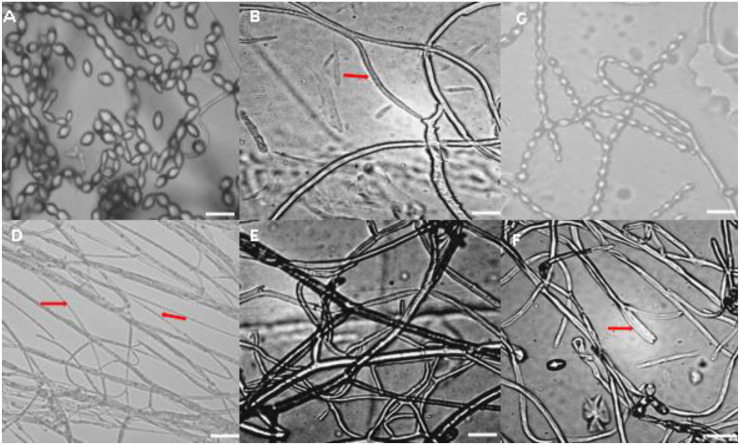
Microscopic observation of hyphal abnormalities of *M. fructigena* after 15 days of incubation at 25 °C. Red arrows indicated mycelial deformations; (**A**) Untreated control (normal hyphae with conidia); (**B**) 2.5% Sodium hydroxide (anormal hyphae); (**C**) 2% Magnesium chlorite (normal hyphae with sporulation); (**D**) 5% Copper sulphate (fine and degraded mycelium); (**E**) 2% Ammonium carbonate (abnormal mycelia structure); and (**F**) 2.5% Sodium carbonate 0.5% (degraded mycelium) (Scale bar = 25 μm), 400× *g* magnification.

After 15-day incubation periods, microscopic observation of the mycelium of the treated pathogen was performed and, indeed, alterations and anomalies were observed in the mycelium form of *M. fructigena*, with changes in mycelial structures due to the different salt treatments (Figure 2).

### 3.3. Effect of Food Preservatives on Spore Germination and Germ Tube Elongation

#### 3.3.1. Effect of Treatments on Spore Germination of *M. fructigena*

The results indicate a significant inhibition of spore germination by most salt treatments (Table 3). Spore germination was completely inhibited at a 5% concentration of sodium carbonate, copper sulphate, sodium hydroxide, and ammonium bicarbonate. These results were similar to those obtained with the fungicidal treatment difenoconazole (1 ppm), which displayed 100% inhibition. In addition, magnesium sulphate exhibited the lowest inhibition rate (25.74%) at a concentration of 0.5%, while sodium phosphate and magnesium chlorite displayed an inhibition rate of 21.94 and 34.60%, respectively.

**Figure 3:**
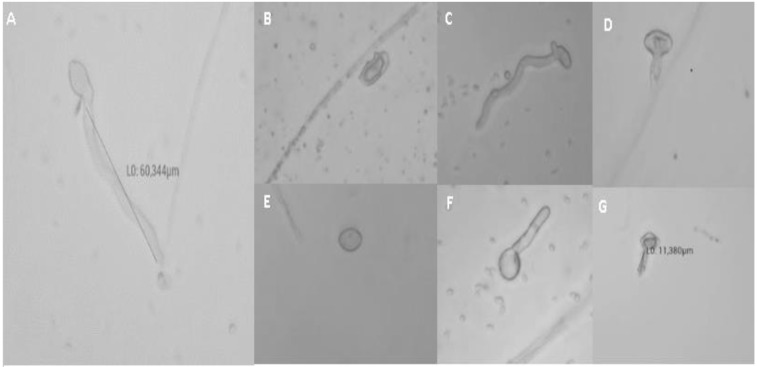
Microscopic observations of spores of *M. fructigena* after 24 h of incubation at 25 °C with agitation. Normal and germinated spore in untreated control (**A**); Ammonium bicarbonate (5%) (**B**); Magnesium chlorite (0.5%) (**C**); Sodium bicarbonate (0.5%) (**D**); Copper sulphate ((**E**), 2.5%); Sodium phosphate dibasic ((**F**), 2%); and Magnesium sulphate ((**G**), 2%).

The shape of the spores differs from one treatment to another (Figure 3). There was either the appearance of a long or normal germ tube for the untreated control or the appearance of a short germ tube with some anomalies and deformation of the spore under salt treatments.

#### 3.3.2. Effect of Salt Treatments on Germ Tube Elongation of *M. fructigena*

The results presented in Table 4 reveal a significant effect of salt treatments on the germ tube elongation of *M. fructigena*. Interestingly, some additives showed a substantial reduction of the germ tube elongation of *M. fructigena* after 24 h of incubation at 25 °C. Sodium carbonate and copper sulphate exhibited remarkable inhibitory effects, leading to the complete inhibition of germ tube formation at concentrations of 2.5% and 5%. These results are comparable to the efficacy of the fungicidal treatment difenoconazole, which was applied at a concentration of 1 ppm. On the other hand, the ammonium bicarbonate has reduced the germ tube elongation of the pathogen by 92.40% at 0.5 g/100 mL and by 100% at 5 g/100 mL. It was concluded that the germ tube length of the pathogen spores indeed varies according to the salt treatment and its concentration. Importantly, these findings seem to be correlated positively with the inhibition rate of germinated spores.

### 3.4. In Vivo Effectiveness of Food Additives against Brown Rot Disease

#### 3.4.1. Food Additive Effects on Disease Severity of *M. fructigena* in Apple Fruit

**Figure 4:**
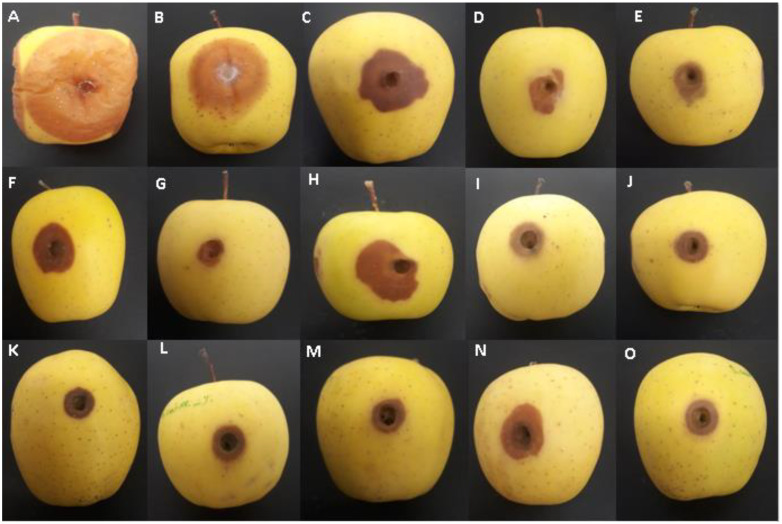
The symptoms of brown rot disease on apple fruit as affected by different salt treatments at 22 °C and 10 days post-inoculation. (**A**) Untreated control, (**B**) Potassium silicate 0.5%; (**C**) Ammonium carbonate 0.5%; (**D**) Ammonium bicarbonate 0.5%; (**E**) Sodium hydride 2%; (**F**) Sodium phosphate dibasic 2%; (**G**) Ammonium bicarbonate 2%; (**H**) Sodium acetate 2%; (**I**) Sodium carbonate 2%; (**J**) Sodium hydroxide 2.5%; (**K**) Ammonium bicarbonate 2.5%; (**L**) Copper sulphate 2%; (**M**) Sodium carbonate 2.5%; (**N**) Magnesium sulphate 2.5%; and (**O**) Copper sulphate 2.5%.

The treatment of apple fruit with food additive compounds as an alternative to conventional fungicides has significantly reduced the development of the brown rot disease caused by *M. fructigena* on artificially infected apples when compared to the untreated control (Figure 4 and Table 5). A significant variation in efficacy between different food additives was observed. In general, food additives highly decreased the severity of the disease. The treatments with sodium carbonate and copper sulphate significantly decreased the severity of the pathogen (100%). This reduction was 6.38% for magnesium sulphate and 100% for ammonium bicarbonate. The fruit wall of the apple fruit was damaged around the wound and the severity of this alteration was dependent on the salt treatment. Indeed, the fruit did not show an intense rot for copper sulphate after 10 days of incubation, as shown in Figure 4.

#### 3.4.2. Food Additive Effects on the Incidence of *M. fructigena* in Apple Fruit

The statistical analysis of the results obtained revealed that sodium carbonate at concentrations of 5% reduced the severity of brown rot up to 100% after 5 days of the incubation period (Table 6). However, when applied at 2%, the sodium hydroxide showed an incidence of 33.33% after 5 days of incubation, while ammonium bicarbonate suppressed the occurrence of the disease in artificially wounded and inoculated apple fruit regardless of the applied concentration (2, 2.5, and 5%) with 100% inhibition (0% severity), which was similar to that of fungicidal difenoconazole at 1ppm (1 µg/mL).

### 3.5. Effect of Preventive Applications on the Quality of Treated Apple Fruit

The inhibition rates of mycelial fungal growth were relatively higher with the food additives sodium bicarbonate, sodium carbonate, copper sulphate, sodium hydroxide, and ammonium bicarbonate than with other additives, and exceeded 88% for most of the concentrations tested. Therefore, these additives were used for the in vivo trials, to evaluate apple fruit quality parameters, and for the semi-commercial trial, while other additives were not considered in the subsequent in vivo trials.

#### 3.5.1. Weight Loss (%)

The results shown in Table 7 reveal that treatments with five food additives had a significant effect on the reduction in weight loss (%). All treatments have a lower weight loss than the untreated control (7.29%), except for sodium hydroxide, which showed a reduction like the untreated control (7.40%) at a concentration of 2%. In addition, their effectiveness in reducing weight loss increased with increasing the concentrations of the salts. For apples inoculated with *M. fructigena*, sodium bicarbonate, sodium carbonate, and copper sulphate showed the highest reduction rates in weight loss with 0.46, 1.94, and 0.91%, respectively, at a concentration of 5%.

#### 3.5.2. Total Soluble Solid Content (TSS)

TSS was determined for all treatments (Table 8). In general, the TSS values in infected apples with *M. fructigena* decreased slightly. Indeed, the Brix of the fruit varied from 12.1 to 14.90% and it is significantly affected by salt treatments. The ammonium bicarbonate at 0.5% shows no significant difference from the untreated control with a Brix value of 15.16%.

#### 3.5.3. Fruits Firmness (N)

The evaluation of fruit firmness indicated that all tested food additives positively affected the preservation of fruit firmness. In addition, the results showed a significant effect of salt treatments on fruit firmness (Table 9). The highest protective activity was shown with sodium hydroxide with an average of 7.48 N, while sodium carbonate at 0.5% showed a firmness of 7.26 N. The lowest value (1.80 N) was obtained with 0.5% of sodium bicarbonate.

#### 3.5.4. Malic Acid (MA)

The measurement of the malic acid level revealed a significant effect of salt treatments. The titratable acidity increased proportionally to the concentration of food additives after 10 days of incubation at 22 °C. The level of MA in the untreated apple control was lower than that recorded in the treated apple fruit (Table 10).

#### 3.5.5. Maturity Index

Regarding the maturity index, significant variations in MI between treatments were observed (Table 11). Apple fruit inoculated with *M. fructigena* and treated with sodium carbonate at 2.5% had the lowest maturity index when compared to other food additives, and reached 5.05. In addition, the maturity index varied according to the food additive and concentration used. The maturity index was 27.67 in control treatments and around 6.61 in fungicidal treatment.

### 3.6. Treatements’ Effect in Large-Scale Semi-Commercial Trials

**Figure 5:**
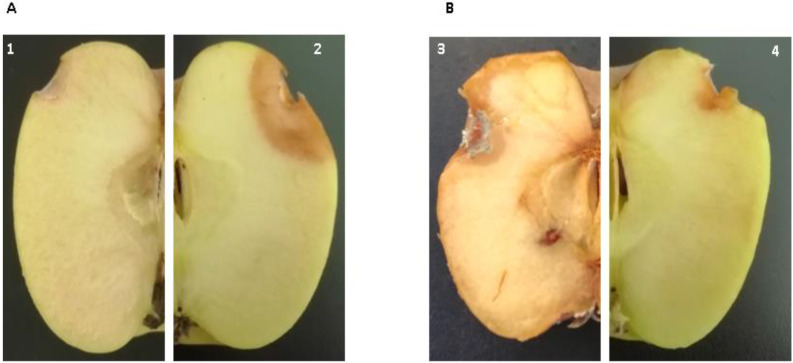
Apple fruit infected by *M. fructigena* (4 × 10^4^ spores/mL) and treated with different treatments and at two temperatures, 4 °C and 22 °C. (**A**) apple fruit at 4 °C (1: Ammonium bicarbonate at 2.5% after 10 days of incubation and 2: Untreated fruit after 10 days at 4 °C) and (**B**) Apple fruit at 22 °C (3: Untreated fruit after 20 days and 4: Sodium carbonate at 2% after 20 days).

Results obtained in the semi-commercial trail at two different temperatures and different incubation periods are listed in Table 12 and Table 13. Regardless of the incubation temperature, apple fruit infected with the fungal pathogen and treated with selected food additives showed lower incidence in comparison to the untreated control (100%). Salt treatments gave incidences varying from 33.33 to 100%. The symptoms of brown rot disease were absent 5 days post-inoculation and started to appear after 10 days with an incidence ranging from 33.33 to 100%. Interestingly, the disease severity of brown rot was dependent on the salt treatment and incubation temperature (Figure 5).

## 4. Discussion

In recent years, there has been a growing recognition of the significance of employing food additives for the safeguarding of fresh fruits from spoilage [38]. The postharvest application of these additives offers a potential alternative to the use of chemical fungicides, thereby catering to the demand for organic or “no-residue” fresh fruit. This is particularly important given the current emphasis on reducing fungicide usage [39]. Brown rot, a fungal disease, poses a significant threat to fruit production as it can cause considerable losses. This disease affects fruits both in orchards, with losses ranging from 50% to 75%, and during postharvest stages such as transportation and storage, leading to additional losses of 25% to 50% [39].

This study has provided evidence that specific food additives possess fungistatic properties, effectively inhibiting the germination of conidia and the growth of mycelium associated with brown rot caused by the pathogen *M. fructigena*. These findings suggest the potential utilization of these additives as an alternative to chemical treatments. Specifically, our study revealed that various food additives, when applied at different doses, can effectively suppress the growth of *M. fructigena* in both an in vitro culture medium and artificially inoculated apples. In the in vitro experiments, some food preservatives exhibited remarkable inhibitory effects on the mycelial growth of the pathogen, with inhibition rates exceeding 88%. Notable examples include sodium bicarbonate, sodium carbonate, and sodium hydroxide [39]. However, specific additives such as ammonium bicarbonate, copper sulfate, and magnesium chlorite did not demonstrate a significant reduction in the disease when tested on Petri dishes.

Several growth tests have been conducted to investigate how specific elements affect the development and growth of pathogens and the mycelial form of hyphae in a specific fungus. Under controlled conditions, the mycelium in the control dishes displayed typical characteristics and produced spores after 15 days of incubation. However, it was observed that certain salts, such as copper sulphate, exhibited the ability to destroy or deform the pathogen’s mycelium, rendering it finer and weaker.

Previous studies have highlighted the effectiveness of carbonate salts against various postharvest fungi affecting fruits and vegetables [39,40]. Similarly, SMBS and PMBS salts demonstrated significant efficacy in inhibiting the growth of *G. citri-aurantii*, *P. italicum*, and *P. digitatum*, with all tested concentrations completely inhibiting their growth after 7 days of incubation at 25 °C [41]. Furthermore, our findings indicated that sodium bicarbonate (4%) completely inhibited the mycelial growth and spore germination of Aspergillus in an in vitro assay [42]. Other salts and food additives, such as ammonium bicarbonate, ammonium carbonate, sodium bicarbonate, sodium carbonate, potassium bicarbonate, and potassium carbonate, exhibited enhanced potential to inhibit the mycelial growth of *B. cinerea* across all tested concentrations [43]. At certain concentrations, researchers have also discovered that hydrogen peroxide, potassium sorbate, sodium bicarbonate, and chitosan can effectively impede the mycelial growth of the *Colletotrichum* sp. strain [43]. Similarly, these salts exhibited the complete inhibition (100%) of the radial mycelial growth of *Colletotrichum siamense*, the anthracnose-causing agent [44].

Carbonate salts have consistently demonstrated effective control over various postharvest fungi in fruits and vegetables, contributing to improved fruit firmness and reduced decay [45]. However, the precise mode of action of these salts in reducing postharvest diseases is not yet fully understood. It is suggested that their inhibitory effects may be attributed to the presence of salt residues in the infection sites occupied by the pathogen [46]. Additionally, the strong inhibitory effect of these substances on pathogenic mycelial growth underscores their potential as a viable strategy for managing postharvest diseases. Many coating formulations incorporate salts, such as bicarbonates and parabens, which have shown significant reduction rates in the brown rot incidence caused by *M. fructicola*. Notably, a coating formulation containing 1.0% potassium sorbate exhibited the highest effectiveness with a reduction rate of 28.6% [26].

The investigation of various treatments on the interaction with pathogen spores yielded diverse outcomes. Notably, copper sulphate, sodium bicarbonate, ammonium bicarbonate, and sodium carbonate exhibited a successful inhibition of *M. fuctigena* spore germination, with inhibition rates ranging from 21.94% for sodium phosphate dibasic to 100% for copper sulphate. Conversely, other salts demonstrated only weak inhibitory effects on germination [42].

In a separate study, the application of SMBS and PMBS significantly reduced the incidence, severity, and sporulation of blue mold and green mold caused by *P. digitatum*, *P. italicum*, and *G. citriaurantii* on ‘Valencia’ oranges, as compared to a control group [42]. Another study found that ammonium carbonate completely inhibited spore germination at a concentration of 10 mM [46]. Furthermore, treatment with hydrogen peroxide and potassium sorbate demonstrated the inhibition of conidia germination in *Colletotrichum* sp. [47].

Food additives have proven effective against a wide range of pathogens. For instance, a preharvest application of potassium phosphate significantly reduced the incidence of citrus brown rot caused by *Phytophthora* spp., with incidences decreasing by 40–60% in harvested and inoculated lemons for up to 75 days post-treatment [45]. Additionally, various salts such as Na_2_CO_3_, NaClO, NaHCO_3_, CaCl_2_, and NaCl completely inhibited conidial germination in banana-crown-rot-causing fungal pathogens such as *Lasiodiplodia theobromae*, *Colletotrichum musae*, *Thielaviopsis paradoxa*, and *Fusarium verticillioides* for 2 days. These salts also displayed complete control of conidial germination in all pathogens for 7 days when supplemented with a surfactant [48]. Furthermore, the treatments exhibited noticeable effects on the germination and germ tubes of the pathogen. While the spores did germinate, their germ tubes were significantly reduced compared to the control group. Sodium bicarbonate achieved the highest reduction, reaching up to 95.53% after 24 h of incubation at 25 °C. Conversely, microscopic examination of conidia treated with substances that displayed high germination inhibition rates revealed the complete absence of germ tubes and occasional spore deformation, as observed with spores treated with 5% copper sulphate.

A series of in vivo tests were conducted to evaluate the effectiveness of various food preservatives in preventing the occurrence and severity of disease in artificially infected apple fruits. Results indicated that the treatments differed significantly in their ability to inhibit the disease. Specifically, primary screenings revealed that ammonium bicarbonate treatments demonstrated a reduction in disease incidence by as much as 66.67% after 5 days of incubation, while other treatments such as magnesium chlorite showed negligible effects compared to untreated fruits.

Further experimentation demonstrated that a combination of copper sulphate, ammonium bicarbonate, sodium hydroxide, sodium carbonate, and sodium bicarbonate treatments administered 24 h before infection significantly reduced the incidence and severity of disease on apples. These findings suggest that certain food preservatives can be effective in preventing and reducing the severity of disease in apples.

Several salts, including sodium benzoate and sodium methylparaben, have shown improvement as alternative methods for controlling sour rot caused by *Geotrichum citri-auranti*. These salts have demonstrated curative effects by reducing the incidence and severity of the disease [49]. Numerous studies have highlighted the potential of various organic and inorganic salts classified as food additives or generally recognized as safe (GRAS) compounds. Examples include potassium silicate, sodium methylparaben, potassium sorbate, and sodium benzoate, which have been effective in controlling various postharvest diseases such as green mold and blue mold [50]. However, the application of copper sulfate has shown phytotoxic effects on fruit rinds, leading to visible darkening, and sinking at the inoculation point. Similar observations were made by Martínez-blay et al. [42], who discovered the phytotoxicity of sodium metabisulfite (SMBS), potassium metabisulfite (PMBS), aluminum sulfate, and aluminum potassium sulfate on citrus fruit [42]. In contrast, the treatment of naturally infected fruits with a 4% solution of sodium bicarbonate successfully controlled the disease caused by *Aspergillus*, achieving a 100% control rate and extending the storage life to 28 days [43]. Furthermore, the combination of these food additives has shown promising results in reducing postharvest diseases, including decaying disease and browning in various fruits. Whangchai et al. [51] reported that ozone, when combined with oxalic or citric acid, could serve as a partial alternative to sulfur dioxide fumigation for controlling decay disease.

In a recent study, the most effective treatments for various diseases, without the need for additional heat treatments, were found to be either potassium sorbate or a sequence of hydrogen peroxide followed by potassium phosphate [52]. Postharvest applications of sodium bicarbonate (SBC) have also been shown to potentially control postharvest diseases in a wide range of fruits, including sweet cherries [53]. Recognizing its significant control potential, sodium was combined with *Metschnikowia fructicola* and ethanol to manage postharvest diseases in grapes [54]. Additionally, researchers have suggested that preharvest calcium treatment, when combined with specific storage atmospheres and fruit injury management, could significantly influence postharvest decay caused by *M. fructigena* [38].

Scientific investigations demonstrated the favorable outcomes of applying cupric salts, specifically copper hydroxide, to cocoa trees to control the brown rot in cocoa pods [55]. In a comprehensive three-year study, the preharvest application of calcium salts on apple fruits revealed a reduction in postharvest losses caused by *M. fructigena*, thereby minimizing the long-term risks of contamination, such as fruit injury and infection [38]. Furthermore, research results indicated that these salts exhibit a direct antifungal effect on *P. digitatum* and possess the ability to induce citrus defense mechanisms against postharvest rot [13]. In the context of controlling anthracnose, the effectiveness of various salts was assessed, with ammonium carbonate (3%) followed by sodium carbonate (2%), either alone or in combination with other salts, demonstrating positive effects in reducing the occurrence of *C. gloeosporioides* in both naturally and artificially inoculated fruits [56].

Subsequently, food additives that exhibited both a low severity rate and an inhibition rate of mycelial growth exceeding 88% in in vitro experiments were selected for testing on apple fruit quality parameters after 15 days of artificial incubation. Employing a preventive approach, the selected treatments were applied within 24 h before inoculation with the bacterial pathogen’s suspension. Remarkably, our study confirmed that the chosen treatments did not compromise the quality of the apples after testing.

Furthermore, the application of sodium bicarbonate as a treatment exhibited various beneficial effects on the quality of yellow pitahaya (*Selenicereus megalanthus*) during both storage and subsequent shelf life [57]. These effects included reduced weight loss, preserved color, and firmness, as well as slowed changes in total soluble solids, titratable acid content, and pH. Another approach involved treating mango fruit with a combination of salicylic acid or potassium phosphonate and a fruit dip containing aqueous sodium bicarbonate, resulting in maintained quality attributes such as pH, total soluble solids, titratable acidity, firmness, and color [58]. The observed outcomes can be attributed to the specific treatments applied, as the storage temperature did not significantly impact the fruit quality. It is noteworthy that other researchers have also conducted experiments under similar storage conditions, supporting these findings. Frans et al. [59] demonstrated that bell pepper cultivars, artificially infected and stored under modified atmosphere packaging conditions, maintained relatively stable levels of total titratable acid, total soluble solids, and vitamin C concentrations for up to 14 days. These conditions were designed to resemble unrefrigerated shelf-life conditions at a challenging temperature of 20 °C.

Over the past two years, numerous studies have been conducted to investigate the impact of storage conditions in conjunction with other treatments on postharvest decay and disease in fruits and vegetables. For instance, in the case of Embul bananas, storage at cold temperatures for up to 21 days was found to effectively control postharvest decay [60]. Similarly, storing Keitt mangoes at 7 °C extended their lifespan for up to six weeks, while storage at 13 °C was shown to be optimal for preserving their quality for up to 21 days without causing chilling injury [61]. Salicylic acid treatments applied during cold storage also significantly improved the storage life of Chimarrita peaches [62]. In addition, modified atmosphere packaging was found to be a valuable tool in preventing postharvest internal rotting of bell peppers caused by *Fusarium* spp. under conventional storage temperatures of 7–16 °C [59].

Storage temperature is a crucial factor in controlling postharvest fungal disease, as demonstrated by a recent study on the effect of temperature on the volatile organic compound (VOC) profile of garlic [63]. For instance, hexanal, calcium chloride, and cold storage are recommended to maintain the quality of mango fruit [64]. Additionally, the use of *Bacillus subtilis* strain QST 713 in the alternative fight against postharvest disease in greenhouse tomatoes was found to be effective when combined with storage at 13 °C for no more than 12 days [65]. On the other hand, a study on the storage of potatoes found no significant difference in the effectiveness of different temperatures [65].

## 5. Conclusions

To summarize, the inhibitory effects of food additives were examined both in vitro and in vivo as control agents against *M. fructigena*, resulting in significant differences. Notably, ammonium bicarbonate, sodium bicarbonate, sodium hydroxide, and copper sulphate exhibited superior efficacy as control agents in both experimental settings. These substances effectively suppressed the incidence and severity of brown rot disease in apples. However, notable variations were observed between the in vitro and in vivo experiments in terms of the antimicrobial efficacy of all the control agents. These discrepancies can be attributed to the differing compositions of each element. During the in vivo testing, the agents were able to gradually disperse across the fruit surface, interacting with the pathogen’s spores and thereby reducing the risk of fruit contamination by *M. fructigena*.

This study demonstrated the potential of several food additives as effective antimicrobial control agents against *M. fructigena*. Notably, ammonium bicarbonate, sodium hydroxide, sodium carbonate, sodium bicarbonate, and copper sulphate displayed remarkable inhibitory effects on *M. fructigena* mycelial growth, both on potato dextrose agar and in vivo on apple fruits that were inoculated and treated preventively. However, further research is needed to enhance the application of food additives for postharvest fungal disease control, as the current knowledge in this area remains limited. Consequently, it is imperative to promote scientific investigations and develop appropriate formulations to effectively manage fungal pathogens, representing a key objective for future investigations.

## Figures and Tables

**Table 1:** Characteristics of antimicrobial food additives used as treatments against *M. fructigena*.

**Food Additives**	**Acronym**	**Molecular Formula**	**E-Code ^a^**	**MW ^b^**
Sulphate copper	SC	CuSO_4_	E519	159.609
Ammonium sulphate	AS	(NH_4_)_2_SO_4_	E517	132.14
Potassium carbonate	PC	K_2_CO_3_	E-501	138.21
Potassium silicate	PSi	K_2_SiO_3_	E560	154.26
Anhydrous sodium sulphate	ASS	Na_2_SO_4_	E515	142.04
Potassium hydroxide	PH	KOH	E525	56.1056
Magnesium chlorite	MC	MgCl_2_	E511	95.211
Sodium phosphate dibasic	SPD	Na_2_HPO_4_	E339	141.96
Sodium acetate	SA	CH_3_COONa	E-262	82.03
Magnesium sulphate	SM	MgSO_4_	E518	120.366
Sodium carbonate	SC	Na_2_CO_3_	E500	105.99
Citric acid	CA	C_6_H_8_O_7_	E330	192.124
Sodium bicarbonate	SBC	NaHCO_3_	E-500	84.01
Ammonium carbonate	AC	(NH_4_)_2_CO_3_	E503	114.10
Ammonium bicarbonate	ABC	NH_4_HCO_3_	E503	79.06
Sodium hydroxide	SH	NaOH	E524	39.997

^a^ E-code = code number for food additives approved by the European Union; ^b^ molecular weight.

**Table 2:** Inhibition rate of mycelial growth (%) of *M. fructigena* obtained by the different agents after 5 and 15 days of incubation at 25 °C in darkness.

Food Preservatives	Inhibition Rate of *M. fructigena* (%)							
	Antimicrobial Agent Concentrations							
	0.5		2		2.5		5	
	5 Days	15 Days	5 Days	15 Days	5 Days	15 Days	5 Days	15 Days
Copper sulphate	69.68 ± 0.67 ^o^	75.96 ± 0.94 ^o^	92.90 ± 2.50 ^m^	89.90 ± 2.39 ^n^	93.08 ± 2.53 ^n^	93.46 ± 4.14 ^n^	100.0 ± 0.00 ^n^	100.0 ± 0.00 ^o^
Ammonium sulphate	25.75 ± 6.48 ^e^	16.23 ± 4.50 ^e^	62.73 ± 0.50 ^j^	39.35 ± 0.26 ^e^	54.90 ± 0.67 ^c^	43.86 ± 0.34 ^e^	75.10 ± 1.00 ^j^	66.06 ± 2.06 ^e^
Potassium carbonate	38.26 ± 1.60 ^j^	17.22 ± 3.84 ^f^	52.89 ± 3.54 ^d^	55.48 ± 0.93 ^h^	58.11 ± 0.77 ^f^	66.83 ± 6.77 ^h^	70.40 ± 0.92 ^h^	75.88 ± 1.86 ^h^
Potassium silicate	39.80 ± 0.82 ^k^	32.38 ± 0.82 ^i^	60.25 ± 0.60 ^g^	66.19 ± 0.60 ^i^	87.75 ± 0.16 ^k^	81.87 ± 0.16 ^k^	91.62 ± 0.13 ^k^	89.73 ± 0.13 ^l^
Sodium sulphate anhydro	46.72 ± 1.75 ^m^	41.77 ± 2.36 ^k^	73.83 ± 2.18 ^k^	66.32 ± 0.25 ^j^	57.86 ± 1.41 ^e^	65.33 ± 1.60 ^h^	64.51 ± 0.49 ^e^	77.75 ± 0.53 ^k^
Potassium hydroxide	61.52 ± 0.59 ^n^	42.15 ± 1.88 ^l^	90.85 ± 0.57 ^l^	87.47 ± 2.75 ^m^	88.95 ± 3.89 ^l^	90.31 ± 3.64 ^m^	97.99 ± 1.41 ^l^	97.32 ± 1.74 ^m^
Magnesium chlorite	31.10 ± 3.68 ^g^	31.09 ± 1.32 ^h^	54.51 ± 0.47 ^e^	41.64 ± 2.28 ^f^	63.52 ± 0.13 ^h^	47.62 ± 0.41 ^g^	70.34 ± 0.87 ^h^	74.33 ± 3.39 ^g^
Sodium phosphate dibasic	93.16 ± 0.43 ^p^	93.86 ± 0.43 ^p^	100.0 ± 0.00 ^o^	100.0 ± 0.00 ^p^	100.0 ± 0.00 ^o^	100.0 ± 0.00 ^o^	100.0 ± 0.00 ^n^	100.0 ± 0.00 ^o^
Sodium acetate	12.82 ± 0.42 ^a^	6.84 ± 3.77 ^c^	26.10 ± 2.42 ^a^	7.84 ± 1.73 ^a^	35.45 ± 1.84 ^a^	27.37 ± 3.43 ^a^	61.44 ± 1.42 ^d^	38.78 ± 3.32 ^b^
Magnesium sulfate	37.65 ± 1.46 ^i^	45.54 ± 1.25 ^m^	61.74 ± 0.784 ^i^	67.32 ± 4.32 ^k^	65.68 ± 0.50 ^i^	77.75 ± 0.36 ^j^	69.06 ± 1.12 ^g^	77.50 ± 2.91 ^j^
Sodium carbonate	43.88 ± 0.53 ^l^	61.31 ± 0.94 ^n^	60.40 ± 0.52 ^h^	72.20 ± 2.05 ^l^	66.00 ± 0.70 ^j^	76.22 ± 1.49 ^i^	71.04 ± 0.56 ^i^	76.96 ± 0.42 ^i^
Sodium bicarbonate	28.20 ± 4.09 ^f^	38.11 ± 0.72 ^j^	58.30 ± 1.37 ^f^	41.77 ± 3.09 ^g^	61.00 ± 1.82 ^g^	47.03 ± 2.16 ^f^	68.28 ± 2.31 ^f^	67.13 ± 2.90 ^f^
Citric acid	32.44 ± 3.30 ^h^	22.08 ± 6.49 ^g^	95.357 ± 1.64 ^n^	96.79 ± 2.10 ^o^	90.14 ± 0.11 ^m^	90.04 ± 0.63 ^l^	98.30 ± 1.20 ^m^	97.70 ± 2.88 ^n^
Ammonium carbonate	14.11 ± 2.44 ^b^	0.32 ± 1.13 ^a^	26.11 ± 0.59 ^a^	25.13 ± 2.18 ^c^	42.86 ± 1.06 ^b^	40.14 ± 2.22 ^c^	58.23 ± 0.92 ^b^	50.16 ± 3.78 ^c^
Ammonium bicarbonate	19.15 ± 0.50 ^d^	7.59 ± 1.63 ^d^	29.40 ± 3.67 ^b^	11.06 ± 1.53 ^b^	42.85 ± 0.75 ^b^	38.75 ± 3.07 ^b^	41.16 ± 0.55 ^a^	35.75 ± 2.82 ^a^
Sodium hydroxide	17.72 ± 1.41 ^c^	5.33 ± 5.10 ^b^	30.80 ± 3.88 ^c^	38.64 ± 0.41 ^d^	57.64 ± 0.83 ^d^	41.64 ± 2.52 ^d^	59.70 ± 0.27 ^c^	52.91 ± 2.99 ^d^
Difenoconazole (1ppm)	100.0 ± 0.00 ^q^	100.0 ± 0.00 ^q^	100.0 ± 0.00 ^o^	100 ± 0.00 ^p^	100.0 ± 0.00 ^o^	100.0 ± 0.00 ^o^	100.0 ± 0.00 ^n^	100.0 ± 0.00 ^o^

Values are the means of two trials over time with three replicates. Means in columns with letters are significantly different at *p* < 0.05, applied after an ANOVA test.

**Table 3:** Inhibition rate (%) of spore germination of *M. fructigena* as affected by food additive treatments 24 h post-incubation at 25 °C in darkness with agitation.

Food Preservatives	Inhibition Rate of Spore Germination of *M. fructigena* (%)			
	Antimicrobial Agent Concentrations			
	0.5	2	2.5	5
Ammonium bicarbonate	78.06 ± 4.18 ^n^	91.14 ± 2.07 ^m^	96.20 ± 3.10 ^k^	100.0 ± 0.00 ^n^
Ammonium carbonate	61.60 ± 3.91 ^g^	55.27 ± 4.88 ^d^	75.53 ± 1.19 ^f^	77.21 ± 1.79 ^e^
Ammonium sulphate	71.31 ± 4.77 ^j^	67.09 ± 3.73 ^e^	75.53 ± 1.19 ^f^	78.90 ± 3.16 ^f^
Sodium bicarbonate	61.18 ± 1.19 ^f^	74.26 ± 1.58 ^h^	88.18 ± 0.60 ^j^	85.23 ± 2.98 ^i^
Potassium carbonate	72.99 ± 1.19 ^k^	74.68 ± 1.03 ^i^	78.90 ± 1.19 ^g^	89.87 ± 2.73 ^l^
Sodium carbonate	75.95 ± 1.03 ^l^	86.50 ± 2.15 ^l^	100.0 ± 0.00 ^l^	100.0 ± 0.00 ^n^
Citric acid	67.09 ± 4.13 ^h^	71.31 ± 3.91 ^f^	74.68 ± 2.07 ^e^	81.43 ± 0.60 ^h^
Copper sulphate	78.90 ± 2.98 ^o^	91.98 ± 5.69 ^n^	100.0 ± 0.00 ^l^	100.0 ± 0.00 ^n^
Magnesium chlorite	34.60 ± 2.98 ^c^	46.41 ±9.26 ^b^	70.88 ± 5.37 ^d^	69.62 ± 4.74 ^d^
Potassium hydroxide	60.76 ± 1.79 ^e^	73.42 ± 2.73 ^g^	88.18 ± 1.58 ^j^	81.01 ± 4.13 ^g^
Potassium silicate	67.51 ± 6.88 ^i^	78.48 ± 6.45 ^k^	83.54 ± 2.73 ^h^	88.18 ± 0.60 ^k^
Sodium acetate	67.51 ± 5.30 ^i^	75.53 ± 2.39 ^j^	84.81 ± 2.73 ^i^	86.92 ± 3.63 ^j^
Sodium hydroxide	76.79 ± 2.98 ^m^	100.0 ± 0.00 ^o^	95.78 ± 3.63 ^k^	97.47 ± 3.58 ^m^
Sodium phosphate dibasic	21.94 ± 3.91 ^a^	48.10 ± 1.79 ^c^	56.96 ± 4.74 ^a^	64.13 ± 1.58 ^b^
Sodium sulfate anhydrous	37.55 ± 4.18 ^d^	41.35 ± 2.98 ^a^	62.02 ± 2.73 ^c^	68.78 ± 3.91 ^c^
Magnesium sulphate	25.74 ± 5.67 ^b^	41.35 ± 1.58 ^a^	61.60 ± 2.60 ^b^	61.18 ± 3.91 ^a^
Difenoconazole (1 ppm)	100.0 ± 0.00 ^p^	100.0 ± 0.00 ^o^	100.0 ± 0.00 ^l^	100.0 ± 0.00 ^n^

Values are means of two trials over time with three replicates. Means in columns with different letters are significantly different at *p* < 0.05, applied after an ANOVA test.

**Table 4:** Food preservative effect on germ tube length of *M. fructigena* was recorded after 24 h at 25 °C.

Food Preservatives	Spore Elongation of *M. fructigena* (%)			
	Antimicrobial Agent Concentrations			
	0.5	2	2.5	5
Ammonium bicarbonate	92.40 ± 0.60 ^p^	95.91 ± 0.45 ^l^	95.85 ± 3.20 ^m^	100.0 ± 0.00 ^n^
Ammonium carbonate	64.26 ± 3.67 ^e^	76.29 ± 0.07 ^h^	79.22 ± 0.31 ^i^	76.97 ± 1.18 ^g^
Ammonium sulphate	69.47 ± 0.397 ^i^	76.59 ± 0.50 ^i^	79.46 ± 0.83 ^j^	74.02 ± 0.45 ^f^
Sodium bicarbonate	76.79 ± 1.29 ^l^	94.36 ± 0.13 ^k^	95.53 ± 0.73 ^l^	95.03 ± 1.02 ^l^
Potassium carbonate	64.97 ± 2.15 ^f^	71.80 ± 0.33 ^f^	77.47 ± 2.04 ^g^	84.54 ± 3.45 ^j^
Sodium carbonate	92.67 ± 0.44 ^p^	96.33 ± 0.51 ^m^	100.0 ± 0.00 ^o^	100.0 ± 0.00 ^n^
Citric acid	69.99 ± 0.09 ^k^	72.16 ± 0.85 ^g^	77.24 ± 0.66 ^f^	78.27 ± 1.46 ^h^
Copper sulphate	91.11 ± 0.30 ^o^	97.27 ± 1.94 ^n^	100.0 ± 0.00 ^o^	100.0 ± 0.00 ^n^
Magnesium chlorite	4.22 ± 0.89 ^a^	18.07 ± 1.02 ^a^	33.08 ± 0.95 ^b^	54.34 ± 3.95 ^b^
Potassium hydroxide	69.56 ± 0.45 ^j^	76.77 ± 0.39 ^j^	79.12 ± 0.44 ^h^	79.75 ± 0.78 ^i^
Potassium silicate	65.30 ± 1.15 ^g^	76.76 ± 0.67 ^j^	82.16 ± 0.46 ^k^	85.30 ± 2.62 ^k^
Sodium acetate	67.75 ± 0.13 ^h^	69.77 ± 0.02 ^e^	72.23 ± 0.77 ^e^	72.83 ± 0.43 ^e^
Sodium hydroxide	88.36 ± 0.53 ^n^	100.0 ± 0.00 ^o^	96.53 ± 2.46 ^n^	98.58 ± 2.01 ^m^
Sodium phosphate dibasic	5.86 ± 4.54 ^b^	35.39 ± 0.54 ^d^	44.54 ± 2.34 ^c^	50.86 ± 2.46 ^a^
Sodium sulphate anhydrous	14.32 ± 0.34 ^d^	29.43 ± 0.78 ^c^	45.48 ± 2.98 ^d^	58.51 ± 0.47 ^d^
Magnesium sulphate	7.38 ± 5.15 ^c^	19.15 ± 4.16 ^b^	31.46 ± 2.95 ^a^	54.90 ± 5.45 ^c^
Difenoconazole (1 ppm)	100.0 ± 0.00 ^q^	100.0 ± 0.00 ^o^	100.0 ± 0.00 ^o^	100.0 ± 0.00 ^n^

Values of the same column followed by the letters within each column are statistically different at *p* < 0.05 according to the ANOVA test.

**Table 5:** Disease severity (DS %) of brown rot on artificially wound-inoculated apple fruit (4 mm wounds; *M. fructigena* at 4.5 × 104 conidia/mL) obtained with food additives after 5 and 10 days of incubation at 22 °C.

Food Preservatives	Severity of *M. fructigena* DS (%)							
	Antimicrobial Agent Concentrations							
	0.5		2		2.5		5	
	5 Days	10 Days	5 Days	10 Days	5 Days	10 Days	5 Days	10 Days
Copper sulphate	88.44 ± 2.45 ^p^	75.40 ± 0.14 ^m^	100.0 ± 0.00 ^l^	96.38 ± 2.05 ^p^	100.0 ± 0.00 ^k^	92.79 ± 2.23 ^m^	100.0 ± 0.00 ^c^	100.0 ± 0.00 ^l^
Ammonium sulphate	45.09 ± 0.87 ^j^	47.88 ± 1.06 ^i^	49.0 ± 0.90 ^d^	53.14 ± 0.53 ^d^	80.78 ± 4.08 ^g^	71.14 ± 5.02 ^e^	85.47 ±3.08 ^a^	84.66 ± 4.36 ^c^
Potassium carbonate	26.2 ± 0.11 ^e^	23.72 ± 1.07 ^g^	100.0 ± 0.00 ^l^	92.31 ± 4.35 ^m^	52.18 ± 0.26 ^c^	66.58 ± 3.18 ^d^	86.16 ± 2.94 ^b^	72.45 ± 7.80 ^a^
Potassium silicate	59.49 ± 4.30 ^k^	13.05 ± 2.47 ^c^	92.58 ± 1.58 ^i^	70.35 ± 0.61 ^g^	100.0 ± 0.00 ^k^	91.47 ± 4.82 ^l^	100.0 ± 0.00 ^c^	100.0 ± 0.00 ^l^
Sodium sulphate anhydro	8.38 ± 0.32 ^b^	20.85 ± 6.14 ^f^	20.0 ± 0.82 ^b^	26.84 ± 0.63 ^a^	48.13 ± 5.52 ^b^	51.75 ± 10.27 ^a^	100.0 ± 0.00 ^c^	88.06 ± 1.26 ^e^
Potassium hydroxide	28.91 ± 0.64 ^h^	49.25 ± 0.23 ^j^	80.27 ± 2.10 ^f^	65.55 ± 6.21 ^f^	83.11 ± 3.58 ^h^	84.25 ± 5.04 ^h^	100.0 ± 0.00 ^c^	96.2 ± 2.15 ^k^
Magnesium chlorite	7.62 ± 0.46 ^a^	15 ± 4.55 ^d^	28.38 ± 0.53 ^c^	39.38 ± 2.66 ^b^	38.55 ± 0.60 ^a^	52.52 ± 0.01 ^b^	100.0 ± 0.00 ^c^	83.83 ± 0.32 ^b^
Sodium phosphate dibasic	17.78 ± 1.25 ^c^	11.67 ± 2.62 ^b^	61.95 ± 0.84 ^e^	59.72 ± 4.32 ^e^	100.0 ± 0.00 ^k^	85.73 ± 2.43 ^i^	100.0 ± 0.00 ^c^	87.21 ± 0.16 ^d^
Sodium acetate	28.22 ± 0.33 ^g^	43.97 ± 1.08 ^h^	100.0 ± 0.00 ^l^	97.33 ± 1.51	84.82 ± 1.61 ^i^	82.17 ± 2.64 ^g^	100.0 ± 0.00 ^c^	94.72 ± 1.49 ^h^
Magnesium sulphate	21.56 ± 0.55 ^d^	6.38 ± 3.15 ^a^	15.78 ± 0.63 ^a^	42.95 ± 0.60 ^c^	79.11 ± 4.43 ^f^	85.78 ± 3.77 ^j^	100.0 ± 0.00 ^c^	88.35 ± 0.75 ^f^
Sodium carbonate	84.15 ± 1.70 ^o^	84.92 ± 2.11 ^p^	100.0 ± 0.00 ^l^	94.47 ± 1.73 ^n^	100.0 ± 00 ^k^	96.42 ± 2.03 ^n^	100.0 ± 0.00 ^c^	100.0 ± 0.00 ^l^
Sodium bicarbonate	72.82 ± 2.88 ^m^	80.64 ± 5.50 ^n^	92.89 ± 1.50 ^j^	94.75 ± 1.55 ^o^	76.58 ± 2.48 ^e^	78.52 ± 3.56 ^f^	100.0 ± 0.00 ^c^	100.0 ± 0.00 ^l^
Citric acid	26.22 ± 0.09 ^f^	20.73 ± 1.27 ^e^	81.22 ± 3.98 ^g^	77.84 ± 5.77 ^i^	62.93 ± 0.57 ^d^	64.83 ± 2.22 ^c^	100.0 ± 0.00 ^c^	95.46 ± 2.57 ^j^
Ammonium carbonate	38.75 ± 0.14 ^i^	58.14 ± 4.07 ^k^	92.33 ± 1.63 ^h^	76.88 ± 9.52 ^h^	92.89 ± 1.51 ^j^	88.75 ± 4.36 ^k^	100.0 ± 0.00 ^c^	94.67 ± 3.02 ^g^
Ammonium bicarbonate	81.2 ± 2.00 ^n^	83.75 ± 3.24 ^o^	100.0 ± 0.00 ^l^	90.57 ± 0.55 ^l^	100.0 ± 0.00 ^k^	100.0 ± 00 ^p^	100.0 ± 0.00 ^c^	100.0 ± 0.00 ^l^
Sodium hydroxide	65.64 ± 0.14 ^l^	74.00 ± 0.43 ^l^	93.55 ± 1.37 ^k^	88.32 ± 1.88 ^k^	100.0 ± 0.00 ^k^	97.5 ± 1.41 ^o^	100.0 ± 0.00 ^c^	95.27 ± 1.34 ^i^
Difenoconazole (1 ppm)	100.0 ± 0.00 ^q^	100.0 ± 0.00 ^q^	100.0 ± 0.00 ^l^	100.0 ± 0.00 ^q^	100.0 ± 0.00 ^k^	100.0 ± 0.00 ^p^	100.0 ± 0.00 ^c^	100.0 ± 0.00 ^l^

Values are the means of two trials over time with three replicates. Means in columns with letters are significantly different at p < 0.05, applied after an ANOVA test.

**Table 6:** Disease incidence (DI, %) of brown rot on artificially wound-inoculated apples (4 mm wounds; *M. fructigena* at 4.5 × 10^4^ conidia/mL) obtained with food additives after 5 and 10 days of incubation at 22 °C.

Disease	Disaese Incidence of *M. fructigena* DI (%)							
	Antimicrobial Agent Concentrations							
	0.5		2		2.5		5	
	5 Days	10 Days	5 Days	10 Days	5 Days	10 Days	5 Days	10 Days
Copper sulphate	33.33 ± 0.47 ^b^	100.0 ± 00 ^c^	00.00 ± 0.00 ^a^	33.33 ± 0.47 ^b^	00.00 ± 0.00 ^a^	66.67 ± 0.47 ^c^	00.00 ± 0.00 ^a^	00.00 ± 0.00 ^a^
Ammonium sulphate	100.0 ± 0.00 ^d^	100.0 ± 00 ^c^	100.0 ± 0.00 ^d^	100.0 ± 0.00 ^d^	33.33 ± 0.47 ^b^	100.0 ± 0.00 ^d^	33.33 ± 0.47 ^b^	66.67 ± 0.47 ^c^
Potassium carbonate	100.0 ± 0.00 ^d^	100.0 ± 00 ^c^	00.00 ± 0.00 ^a^	33.33 ± 0.47 ^b^	100.0 ± 0.00 ^d^	100.0 ± 0.00 ^d^	33.33 ± 0.47 ^b^	66.67 ± 0.47 ^c^
Potassium silicate	66.67 ± 0.47 ^c^	100.0 ± 00 ^c^	33.33 ± 0.47 ^b^	100.0 ± 0.00 ^d^	00.00 ± 0.00 ^a^	33.33 ± 0.47 ^b^	00.00 ± 0.00 ^a^	00.00 ± 0.00 ^a^
Sodium sulphate anhydro	100.0 ± 0.00 ^d^	100.0 ± 00 ^c^	100.0 ± 00.0 ^d^	100.0 ± 0.00 ^d^	66.67 ± 0.47 ^c^	100.0 ± 0.00 ^d^	00.00 ± 0.00 ^a^	100.0 ± 0.00 ^d^
Potassium hydroxide	100.0 ± 0.00 ^d^	100.0 ± 00 ^c^	66.67 ± 0.47 ^c^	100.0 ± 0.00 ^d^	33.33 ± 0.47 ^b^	66.67 ± 0.47 ^c^	00.00 ± 0.00 ^a^	33.33 ± 0.47 ^b^
Magnesium chlorite	100.0 ± 0.00 ^d^	100.0 ± 0.00 ^c^	100.0 ± 0.00 ^d^	100.0 ± 0.00 ^d^	33.33 ± 0.47 ^b^	100.0 ± 0.00 ^d^	00.00 ± 0.00 ^a^	100.0 ± 0.00 ^d^
Sodium phosphate dibasic	100.0 ± 0.00 ^d^	100.0 ± 0.00 ^c^	100.0 ± 0.00 ^d^	100.0 ± 0.00 ^d^	00.00 ± 0.00 ^a^	100.0 ± 0.00 ^d^	00.00 ± 0.00 ^a^	100.0 ± 0.00 ^d^
Sodium acetate	100.0 ± 0.00 ^d^	100.0 ± 0.00 ^c^	00.00 ± 0.00 ^a^	33.33 ± 0.47 ^b^	66.67 ± 0.47 ^c^	100.0 ± 00 ^d^	00.00 ± 0.00 ^a^	66.67 ± 0.47 ^c^
Magnesium sulphate	100.0 ± 0.00 ^d^	100.0 ± 0.00 ^c^	100.0 ± 0.00 ^d^	100.0 ± 0.00 ^d^	33.33 ± 0.47 ^b^	100.0 ± 0.00 ^d^	00.00 ± 0.00 ^a^	100.0 ± 0.00 ^d^
Sodium carbonate	66.67 ± 0.47 ^c^	100.0 ± 0.00 ^c^	00.00 ± 0.00 ^a^	66.67 ± 0.47 ^c^	00.00 ± 0.00 ^a^	33.33 ± 0.47 ^b^	00.00 ± 0.00 ^a^	00.00 ± 0.00 ^a^
Sodium bicarbonate	66.67 ± 0.47 ^c^	66.67 ± 0.47 ^b^	33.33 ± 0.47 ^b^	66.67 ± 0.47 ^c^	66.67 ± 0.47 ^c^	100.0 ± 0.00 ^d^	00.00 ± 0.00 ^a^	00.00 ± 0.00 ^a^
Citric acid	100.0 ± 0.00 ^d^	100.0 ± 0.00 ^c^	33.33 ± 0.47 ^b^	100.0 ± 0.00 ^d^	100.0 ± 0.00 ^d^	100.0 ± 0.00 ^d^	00.00 ± 0.00 ^a^	33.33 ± 0.47 ^b^
Ammonium carbonate	100.0 ± 0.00 ^d^	100.0 ± 0.00 ^c^	33.33 ± 0.47 ^b^	66.67 ± 0.47 ^c^	33.33 ± 0.47 ^b^	66.67 ± 0.47 ^c^	00.00 ± 0.00 ^a^	33.33 ± 0.47 ^b^
Ammonium bicarbonate	66.67 ± 0.47 ^c^	100.0 ± 0.00 ^c^	00.00 ± 0.00 ^a^	100.0 ± 0.00 ^d^	00.00 ± 0.00 ^a^	00.00 ± 0.00 ^a^	00.00 ± 0.00 ^a^	00.00 ± 0.00 ^a^
Sodium hydroxide	100.0 ± 0.00 ^d^	100.0 ± 0.00 ^c^	33.33 ± 0.47 ^b^	100.0 ± 0.00 ^d^	00.00 ± 0.00 ^a^	33.33 ± 0.47 ^b^	00.00 ± 0.00 ^a^	66.67 ± 0.47 ^c^
Difenoconazole (1 ppm)	00.00 ± 0.00 ^a^	00.00 ± 0.00 ^a^	00.00 ± 0.00 ^a^	00.00 ± 0.00 ^a^	00.00 ± 0.00 ^a^	00.00 ± 0.00 ^a^	00.00 ± 0.00 ^a^	00.00 ± 0.00 ^a^

Values are the means of two trials over time with three replicates. Means in columns with letters are significantly different at *p* < 0.05, applied after an ANOVA test.

**Table 7:** Effect of food additives on weight loss of apples artificially inoculated during storage at room temperature.

Food Preservatives	Weight Loss (%)			
	Antimicrobial Agent Concentrations			
	0.5	2	2.5	5
Ammonium bicarbonate	4.50 ± 1.62 ^f^	4.05 ± 1.19 ^e^	1.39 ± 0.56 ^b^	1.62 ± 0.59 ^c^
Sodium bicarbonate	1.12 ± 0.32 ^a^	2.62 ± 0.93 ^d^	4.59 ± 1.38 ^f^	0.46 ± 0.32 ^a^
Sodium carbonate	4.36 ± 0.74 ^e^	1.34 ± 0.04 ^a^	1.00 ± 0.38 ^a^	1.94 ± 0.81 ^d^
Copper sulphate	3.01 ± 1.51 ^c^	2.42 ± 0.77 ^c^	1.53 ± 1.02 ^c^	0.68 ± 0.54 ^b^
Sodium hydroxide	3.05 ± 1.82 ^d^	7.40 ± 4.47 ^g^	1.60 ± 1.78 ^d^	4.31 ± 1.98 ^f^
Difenoconazole (1 ppm)	2.26 ± 1.03 ^b^	2.26 ± 1.03 ^b^	2.26 ± 1.03 ^e^	2.26 ± 1.03 ^e^
Untreated control	7.29 ± 3.51 ^g^	7.29 ± 3.51 ^f^	7.29 ± 3.51 ^g^	7.29 ± 3.51 ^g^

Values are the means of two trials over time with three replicates. Means in columns with letters are significantly different at *p* < 0.05, applied after an ANOVA test.

**Table 8:** Effect of food additives on the Brix of apples artificially inoculated during storage at room temperature.

Food Preservatives	TSS (%)			
	Antimicrobial Agent Concentrations			
	0.5	2	2.5	5
Ammonium bicarbonate	14.90 ± 0.14 ^e^	14.00 ± 0.00 ^e^	14.16 ± 0.12 ^e^	12.06 ± 0.09 ^a^
Sodium bicarbonate	14.20 ± 0.16 ^d^	13.80 ± 0.32 ^d^	14.10 ± 0.08 ^d^	14.03 ± 0.04 ^f^
Sodium carbonate	12.60 ± 0.08 ^b^	13.40 ± 0.43 ^c^	12.07 ± 0.09 ^a^	12.10 ± 0.08 ^b^
Copper sulphate	13.80 ± 0.32 ^c^	14.10 ± 0.08 ^f^	14.10 ± 0.08 ^d^	12.50 ± 0.35 ^d^
Sodium hydroxide	14.20 ± 0.28 ^d^	12.60 ± 0.08 ^b^	13.66 ± 0.20 ^c^	12.13 ± 0.18 ^c^
Difenoconazole (1 ppm)	12.53 ± 0.20 ^a^	12.53 ± 0.20 ^a^	12.53 ± 0.20 ^b^	12.53 ± 0.20 ^e^
Untreated control	15.16 ± 0.12 ^f^	15.16 ± 0.12 ^g^	15.16 ± 0.12 ^f^	15.16 ± 0.12 ^g^

Values are the means of two trials over time with three replicates. Means in columns with letters are significantly different at *p* < 0.05, applied after an ANOVA test.

**Table 9:** Effect of food additives on the firmness of apples artificially inoculated during storage at room temperature.

Food Preservatives	Firmness (N)			
	Antimicrobial Agent Concentrations			
	0.5	2	2.5	5
Ammonium bicarbonate	6.15 ± 0.26 ^c^	6.15 ± 0.13 ^a^	7.37 ± 0.10 ^f^	5.89 ± 0.44 ^b^
Sodium bicarbonate	1.80 ± 0.11 ^a^	7.19 ± 0.34 ^d^	7.27 ± 0.05 ^d^	5.53 ± 1.34 ^a^
Sodium carbonate	7.26 ± 0.07 ^f^	6.72 ± 0.09 ^b^	6.80 ± 0.73 ^a^	7.36 ± 0.16 ^f^
Copper sulphate	6.06 ± 1.04 ^b^	7.06 ± 0.20 ^c^	7.01 ± 0.67 ^b^	6.86 ± 0.20 ^c^
Sodium hydroxide	7.02 ± 0.17 ^d^	7.05 ± 0.11 ^c^	7.06 ± 0.30 ^c^	7.48 ± 0.07 ^g^
Difenoconazole (1 pmm)	7.28 ± 0.11 ^f^	7.28 ± 0.11 ^f^	7.28 ± 0.11 ^e^	7.28 ± 0.11 ^e^
Untreated control	7.21 ± 0.12 ^e^	7.21 ± 0.12 ^e^	7.21 ± 0.12 ^d^	7.21 ± 0.12 ^d^

Values are means of two trials over time with three replicates. Means in columns with letters are significantly different at *p* < 0.05, applied after an ANOVA test.

**Table 10:** Effect of food additives on the Brix of apples artificially inoculated during storage at room temperature.

Food Preservatives	MA(g of Malic Acid/L)			
	Antimicrobial Agent Concentrations			
	0.5	2	2.5	5
Ammonium bicarbonate	0.83 ± 0.11 ^b^	1.34 ± 0.00 ^c^	2.18 ± 0.12 ^d^	1.34 ± 0.00 ^b^
Sodium bicarbonate	1.60 ± 2.23 ^d^	1.34 ± 0.00 ^c^	2.68 ± 0.10 ^f^	2.37 ± 0.24 ^f^
Sodium carbonate	0.84 ± 0.13 ^b^	1.45 ± 0.15 ^e^	2.50 ± 0.49 ^e^	1.43 ± 0.12 ^c^
Copper sulphate	1.65 ± 0.12 ^e^	1.07 ± 0.21 ^b^	1.38 ± 0.15 ^b^	2.27 ± 0.21 ^e^
Sodium hydroxide	1.03 ± 0.17 ^c^	1.38 ± 0.22 ^d^	2.03 ± 0.03 ^c^	2.05 ± 0.06 ^d^
Difenoconazole (1 ppm)	2.03 ± 0.03 ^f^	2.03 ± 0.03 ^f^	2.03 ± 0.03 ^c^	2.03 ± 0.03 ^d^
Untreated control	0.58 ± 0.12 ^a^	0.58 ± 0.12 ^a^	0.58 ± 0.12 ^a^	0.58 ± 0.12 ^a^

Values are means of two trials over time with three replicates. Means in columns with letters are significantly different at *p* < 0.05, applied after an ANOVA test.

**Table 11:** Effect of food additives on the Brix of apple’s artificially inoculated during storage at room temperature.

Food Preservation	Maturity Index (MI)			
	Antimicrobial Agent Concentrations			
	0.5	2	2.5	5
Ammonium bicarbonate	18.41 ± 2.82 ^f^	10.44 ± 0.00 ^e^	6.49 ± 0.47 ^c^	9.00 ± 0.07 ^f^
Sodium bicarbonate	8.83 ± 0.10 ^c^	10.29 ± 0.24 ^d^	5.27 ± 0.24 ^b^	5.99 ± 0.73 ^c^
Sodium carbonate	15.28 ± 2.70 ^e^	9.35 ± 1.15 ^b^	5.05 ± 1.19 ^a^	8.52 ± 0.17 ^e^
Copper sulphate	8.38 ± 0.50 ^b^	13.74 ± 2.95 ^f^	10.31 ± 1.13 ^f^	5.53 ± 0.55 ^a^
Sodium hydroxide	14.28 ± 2.40 ^d^	9.38 ± 1.71 ^c^	6.72 ± 0.04 ^e^	5.91 ± 0.23 ^b^
Difenoconazole (1 ppm)	6.61 ± 0.04 ^a^	6.61 ± 0.04 ^a^	6.61 ± 0.04 ^d^	6.61 ± 0.04 ^d^
Untreated control	27.67 ± 7.16 ^g^	27.67± 7.16 ^g^	27.67 ± 7.16 ^g^	27.67 ± 7.16 ^g^

Values are the means of two trials over time with three replicates. Means in columns with letters are significantly different at *p* < 0.05, applied after an ANOVA test.

**Table 12:** Averaged incidence of brown rot disease caused by *M. fructigena* (4 × 10^4^ spores/mL), observed on apple fruit treated with selected effective food additives and after 5-, 10-, 15-, 20-, and 30-day incubation periods at 4 °C.

Treatment	Concentration	Temperature	Storage Time (Days)				
			5	10	15	20	30
Ammonium bicarbonate	0.5	4 °C	00.00 ± 0.00 ^a^	100.0 ± 0.00 ^d^	100.0 ± 0.00 ^d^	100.0 ± 0.00 ^d^	100.0 ± 0.00 ^d^
	2	4 °C	00.00 ± 0.00 ^a^	100.0 ± 0.00 ^d^	100.0 ± 0.00 ^d^	100.0 ± 0.00 ^d^	100.0 ± 0.00 ^d^
	2.5	4 °C	00.00 ± 0.00 ^a^	00.00 ± 0.00 ^a^	66.66 ± 0.47 ^c^	100.0 ± 0.00 ^d^	100.0 ± 0.00 ^d^
	5	4 °C	00.00 ± 0.00 ^a^	00.00 ± 0.00 ^a^	33.33 ± 0.47 ^b^	100.0 ± 0.00 ^d^	100.0 ± 0.00 ^d^
Sodium bicarbonate	0.5	4 °C	00.00 ± 0.00 ^a^	100.0 ± 0.00 ^d^	100.0 ± 0.00 ^d^	100.0 ± 0.00 ^d^	100.0 ± 0.00 ^d^
	2	4 °C	00.00 ± 0.00 ^a^	66.66 ± 0.47 ^c^	66.66 ± 0.47 ^c^	100.0 ± 0.00 ^d^	100.0 ± 0.00 ^d^
	2.5	4 °C	00.00 ± 0.00 ^a^	66.66 ± 0.47 ^c^	100.0 ± 0.00 ^d^	100.0 ± 0.00 ^d^	100.0 ± 0.00 ^d^
	5	4 °C	00.00 ± 0.00 ^a^	00.00± 0.00 ^a^	33.33 ± 0.47 ^b^	100.0 ± 0.00 ^d^	100.0 ± 0.00 ^d^
Sodium carbonate	0.5	4 °C	00.00 ± 0.00 ^a^	100.0 ± 0.00 ^d^	100.0 ± 0.00 ^d^	100.0 ± 0.00 ^d^	100.0 ± 0.00 ^d^
	2	4 °C	00.00 ± 0.00 ^a^	66.66 ± 0.47 ^c^	100.0 ± 0.00 ^d^	100.0 ± 0.00 ^d^	100.0 ± 0.00 ^d^
	2.5	4 °C	00.00 ± 0.00 ^a^	33.33 ± 0.47 ^b^	100.0 ± 0.00 ^d^	100.0 ± 0.00 ^d^	100.0 ± 0.00 ^d^
	5	4 °C	00.00 ± 0.00 ^a^	00.00 ± 0.00 ^a^	100.0 ± 0.00 ^d^	100.0 ± 0.00 ^d^	100.0 ± 0.00 ^d^
Copper sulphate	0.5	4 °C	00.00 ± 0.00 ^a^	100.0 ± 0.00 ^d^	100.0 ± 0.00 ^d^	100.0 ± 0.00 ^d^	100.0 ± 0.00 ^d^
	2	4 °C	00.00 ± 0.00 ^a^	66.66 ± 0.47 ^c^	66.66 ± 0.47 ^c^	100.0 ± 0.00 ^d^	100.0 ± 0.00 ^d^
	2.5	4 °C	00.00 ± 0.00 ^a^	33.33 ± 0.00 ^b^	33.33 ± 0.00 ^b^	100.0 ± 0.00 ^d^	100.0 ± 0.00 ^d^
	5	4 °C	00.00 ± 0.00 ^a^	00.00 ± 0.00 ^a^	33.33 ± 0.00 ^b^	100.0 ± 0.00 ^d^	100.0 ± 0.00 ^d^
Sodium hydroxide	0.5	4 °C	00.00 ± 0.00 ^a^	100.0 ± 0.00 ^d^	100.0 ± 0.00 ^d^	100.0 ± 0.00 ^d^	100.0 ± 0.00 ^d^
	2	4 °C	00.00 ± 0.00 ^a^	100.0 ± 0.00 ^d^	100.0 ± 0.00 ^d^	100.0 ± 0.00 ^d^	100.0 ± 0.00 ^d^
	2.5	4 °C	00.00 ± 0.00 ^a^	66.66 ± 0.47 ^c^	66.66 ± 0.47 ^c^	100.0 ± 0.00 ^d^	100.0 ± 0.00 ^d^
	5	4 °C	00.00 ± 0.00 ^a^	33.33 ± 0.47 ^b^	66.66 ± 0.47 ^c^	100.0 ± 0.00 ^d^	100.0 ± 0.00 ^d^
Difenoconazole (1 ppm)	_	4 °C	00.00 ± 0.00 ^a^	00.00 ± 0.00 ^a^	00.00 ± 0.00 ^a^	00.00 ± 0.00 ^a^	33.33 ± 0.47 ^b^
Untreated control	_	4 °C	100.0 ± 0.00 ^b^	100.0 ± 0.00 ^d^	100.0 ± 0.00 ^d^	100.0 ± 0.00 ^d^	100.0 ± 0.00 ^d^

Values are the means of two trials over time with 10 replicates. In each column, means with the same letters are not significantly different at *p* < 0.05, applied after an ANOVA test.

**Table 13:** Averaged incidence of brown rot disease caused by *M. fructigena* (4 × 10^4^ spores/mL), observed on apple fruit treated by selected effective antimicrobial food agents for different treatments after incubation at 22 °C for 5, 10, 15, 20, and 30 days.

Treatment	Concentration	Temperature	Storage Time (Day)				
			5	10	15	20	30
Ammonium bicarbonate	0.5	22 °C	66.66 ± 0.47 ^c^	100.0 ± 0.00 ^d^	100.0 ± 0.00 ^d^	100.0 ± 0.00 ^d^	100.0 ± 0.00 ^d^
	2	22 °C	00.00 ± 0.00 ^a^	100.0 ± 0.00 ^d^	100.0 ± 0.00 ^d^	100.0 ± 0.00 ^d^	100.0 ± 0.00 ^d^
	2.5	22 °C	00.00 ± 0.00 ^a^	00.00 ± 0.00 ^a^	00.00 ± 0.00 ^a^	66.66 ± 0.47 ^c^	100.0 ± 0.00 ^d^
	5	22 °C	00.00 ± 0.00 ^a^	00.00 ± 0.00 ^a^	00.00 ± 0.00 ^a^	33.33 ± 0.47 ^b^	100.0 ± 0.00 ^d^
Sodium bicarbonate	0.5	22 °C	33.33 ± 0.47 ^b^	66.66 ± 0.47 ^c^	100.0 ± 0.00 ^d^	100.0 ± 0.00 ^d^	100.0 ± 0.00 ^d^
	2	22 °C	66.66 ± 0.47 ^c^	66.66 ± 0.47 ^c^	66.66 ± 0.47 ^c^	66.66 ± 0.47 ^c^	100.0 ± 0.00 ^d^
	2.5	22 °C	66.66 ± 0.47 ^c^	100.0 ± 0.00 ^d^	66.66 ± 0.47 ^c^	100.0 ± 0.00 ^d^	100.0 ± 0.00 ^d^
	5	22 °C	00.00 ± 0.00 ^a^	00.00 ± 0.00 ^a^	00.00 ± 0.00 ^a^	33.33 ± 0.47 ^b^	100.0 ± 0.00 ^d^
Sodium carbonate	0.5	22 °C	66.66 ± 0.47 ^c^	100.0 ± 0.00 ^d^	100.0 ± 0.00 ^d^	100.0 ± 0.47 ^d^	100.0 ± 0.00 ^d^
	2	22 °C	00.00 ± 0.00 ^a^	66.66 ± 0.47 ^c^	66.66 ± 0.47 ^c^	100.0 ± 0.47 ^d^	100.0 ± 0.00 ^d^
	2.5	22 °C	00.00 ± 0.00 ^a^	33.33 ± 0.47 ^b^	33.33 ± 0.47 ^b^	100.0 ± 0.47 ^d^	100.0 ± 0.00 ^d^
	5	22 °C	00.00 ± 0.00 ^a^	00.00 ± 0.00 ^a^	00.00 ± 0.00 ^a^	100.0 ± 0.47 ^d^	100.0 ± 0.00 ^d^
Copper sulphate	0.5	22 °C	33.33 ± 0.47 ^b^	100.0 ± 0.00 ^d^	100.0 ± 0.00 ^d^	100.0 ± 0.00 ^d^	100.0 ± 0.00 ^d^
	2	22 °C	00.00 ± 0.00 ^a^	66.66 ± 0.47 ^c^	66.66 ± 0.47 ^c^	66.66 ± 0.47 ^c^	100.0 ± 0.00 ^d^
	2.5	22 °C	00.00 ± 0.00 ^a^	33.33 ± 0.47 ^b^	33.33 ± 0.47 ^b^	33.33 ± 0.47 ^b^	100.0 ± 0.00 ^d^
	5	22 °C	00.00 ± 0.00 ^a^	00.00 ± 0.00 ^a^	00.00 ± 0.00 ^a^	33.33 ± 0.47 ^b^	100.0 ± 0.00 ^d^
Sodium hydroxide	0.5	22 °C	100.0 ± 0.00 ^d^	100.0 ± 0.00 ^d^	100.0 ± 0.00 ^d^	100.0 ± 0.47 ^d^	100.0 ± 0.00 ^d^
	2	22 °C	33.33 ± 0.47 ^b^	100.0 ± 0.00 ^d^	100.0 ± 0.00 ^d^	100.0 ± 0.47 ^d^	100.0 ± 0.00 ^d^
	2.5	22 °C	00.00 ± 0.00 ^a^	66.66 ± 0.47 ^c^	66.66 ± 0.47 ^c^	66.66 ± 0.47 ^c^	100.0 ± 0.00 ^d^
	5	22 °C	00.00 ± 0.00 ^a^	33.33 ± 0.47 ^b^	33.33 ± 0.47 ^b^	66.66 ± 0.47 ^c^	100.0 ± 0.00 ^d^
Difenoconazole (1 ppm)	_	22 °C	00.00 ± 0.00 ^a^	00.00 ± 0.00 ^a^	00.00 ± 0.00 ^a^	33.33 ± 0.47 ^b^	66.66 ± 0.47 ^c^
Untreated control	_	22 °C	100.0 ± 0.00 ^d^	100.0 ± 0.00 ^d^	100.0 ± 0.00 ^d^	100.0 ± 0.00 ^d^	100.0 ± 0.00 ^d^

Values are the means of two trials over time with 10 replicates. In each column, means with the same letters are not significantly different at *p* < 0.05, applied after an ANOVA test.

## Data Availability

The data presented in this study are available on request from the corresponding author.
